# Plant Bioactives in the Treatment of Inflammation of Skeletal Muscles: A Molecular Perspective

**DOI:** 10.1155/2022/4295802

**Published:** 2022-07-20

**Authors:** Dipanjan Karati, Ryan Varghese, K. R. Mahadik, Rohit Sharma, Dileep Kumar

**Affiliations:** ^1^Poona College of Pharmacy, Bharati Vidyapeeth (Deemed to be) University, Pune, Maharashtra 411038, India; ^2^Department of Rasa Shastra and Bhaishajya Kalpana, Faculty of Ayurveda, Institute of Medical Sciences, Banaras Hindu University, Varanasi 221005, Uttar Pradesh, India

## Abstract

Skeletal muscle mass responds rapidly to growth stimuli, precipitating hypertrophies (increased protein synthesis) and hyperplasia (activation of the myogenic program). For ages, muscle degeneration has been attributed to changes in the intracellular myofiber pathways. These pathways are tightly regulated by hormones and lymphokines that ultimately pave the way to decreased anabolism and accelerated protein breakdown. Despite the lacunae in our understanding of specific pathways, growing bodies of evidence suggest that the changes in the myogenic/regenerative program are the major contributing factor in the development and progression of muscle wasting. In addition, inflammation plays a key role in the pathophysiology of diseases linked to the failure of skeletal muscles. Chronic inflammation with elevated levels of inflammatory mediators has been observed in a spectrum of diseases, such as inflammatory myopathies and chronic obstructive pulmonary disease (COPD). Although the pathophysiology of these diseases varies greatly, they all demonstrate sarcopenia and dysregulated skeletal muscle physiology as common symptoms. Medicinal plants harbor potential novel chemical moieties for a plenitude of illnesses, and inflammation is no exception. However, despite the vast number of potential antiinflammatory compounds found in plant extracts and isolated components, the research on medicinal plants is highly daunting. This review aims to explore the various phytoconstituents employed in the treatment of inflammatory responses in skeletal muscles, while providing an in-depth molecular insight into the latter.

## 1. Introduction

Skeletal muscles account for about 40% of the body's mass and are essential for performing normal physiological activities. This necessitates the need for maintaining skeletal muscle growth, metabolism, and contractile performance throughout life for total body health. However, the decline in muscle performance is frequently associated with increasing age and other morbid conditions [1].

In both developed and emerging countries, noncommunicable diseases are on the rise, and musculoskeletal disorders (MSDs) are no exception [2]. The global burden of MSDs is approximately 1.7 billion individuals, which precedes both disability and even death [3].

In response to the rapid muscular damage and acute exercise stimulation, the skeletal muscle tends to change its size, shape, and/or function, thereby demonstrating remarkable flexibility. Since time immemorial, exercise has been reported to demonstrate a substantial increase in muscle mass and function (reviewed in [4–6]). However, the elderly and patients with muscle morbidities are restrained due to their ailments and physical limitations. This has further facilitated the development of nonexercise and pharmacotherapeutic interventions as a hotspot for research on MSDs.

In morbid conditions such as Type-2 Diabetes Mellitus and obesity, insulin resistance in the skeletal muscles is a major contributor to impaired whole-body glucose clearance. Since antiquity, plants have been a rich source of pharmacologically active phytochemicals that have been employed in a spectrum of ailments. For example, the ethanolic extract of Russian tarragon (*Artemisia dracunculus* L.) has been studied to be a potent blood sugar reducing agent, which concurrently improves insulin sensitivity in congenital as well as synthetically persuaded diabetic murine models [7, 8]. In addition, the tannase-converted green tea extract could potentially be utilized in the treatment regimen of sarcopenia, among other MSDs [9]. Despite the fact that western medicine (allopathy) and surgical interventions have gained acceptance in recent years, the masses still resort to plant-based medicine as it is highly efficacious, considered safe, and devoid of harmful side effects [10]. Researchers and physicians across the globe have developed an armamentarium of medicinal herbs that could potentially alleviate the symptoms associated with MSD-related disorders.

While several articles emphasise on the role of phytochemicals and plant extracts in the alleviation of symptoms associated with MSDs, the prime focus of this article is to summarize and critically evaluate recent studies, findings, and conclusions.

## 2. Insights into Musculoskeletal Disorders

Skeletal muscles make up about 40% of a person's total weight and are essential for good health. Skeletal muscles are in charge of keeping your posture and allowing you to perform your regular tasks. They help with important biological functions, including nutrition sensing, energy metabolism, core temperature regulation, and organ and bone protection. Muscle function deteriorates with age, resulting in decreased mobility. Fall-related injuries, impairment, loss of independence and a considerable increase in mortality in the elderly can all result. Sarcopenia is a term used to describe the progressive loss of muscle mass and function in the elderly and chronically ill due to muscular fiber atrophy and loss. It plays a significant role in the loss of independence, disability, the need for long-term care, and overall mortality. Sarcopenia is a complicated disease with little knowledge of its etiology. MSDs are a group of diseases that primarily affect the bones, joints, muscles, and connective tissues. These ailments are often reported to cause pain and loss of function and are among the most expensive and disabling conditions in the United States [11]. These MSDs could be a product of either genetic predisposition, congenital illness, or an acquired pathological process. Growing bodies of evidence have also associated their pathophysiology with infection, inflammation, degeneration, trauma, impaired development, neoplasia, as well as vascular and/or toxic/metabolic derangements [12]. With over 1.7 billion individuals being affected by MSDs globally, their disability and mortality rates pose a great health concern [2, 3]. The World Health Organization (WHO) ranks MSDs, such as arthritis, osteoarthritis, bone fractures, and back and neck discomfort, as the second most common cause of debility across the globe [13]. Other major MSDs include chronic lower back pain, osteoarthritis of the hip, knee, wrist, and hand, inflammatory arthropathies such as rheumatoid arthritis and psoriatic arthritis, and arthropathies of other joints [12].

Rheumatoid arthritis is a chronic autoimmune disorder characterized by polyarthralgia and joint inflammation. Antibodies that target self-neoepitopes are triggered by inflammatory cascade mechanisms. These mechanisms are allied by chronic arthritic assaults, which further result in the targeting of self-neoepitopes. The arsenal of macrophages, defense immunocytes, and biomarkers elicited in response to the self-epitopes have paved the way for the development of innovative theranostic modalities. These curative methods serve as effective suppressors of these cells and trigger antibodies, thereby curbing the disease progression as well as providing a deeper understanding of the disease etiopathophysiology [14].

Psoriatic arthritis is a chronic inflammatory condition primarily affecting the entheses, joints, and the spine [12]. The inflammatory effect is also profound in other tissues such as nail involvement and dactylitis, where it occurs in concurrence with psoriatic scaly patches (reviewed in [15]). In most cases, skin indications often precede the onset of arthritis; however, in some people, their symptoms appear at the same time. In about 10–15% of the cases, arthritis precedes the onset of these inflammatory patches [16, 17].

Another major social debility is muscular atrophy, which is a decrease in muscle mass and power, as well as the ability to regenerate. This condition could be precipitated by a plenitude of conditions, including blast distress, cachexia, chronic kidney disease (CKD), diabetes, high-speed accidents, immobility, obesity, preexisting orthopedic conditions, malnutrition, metabolic acidosis, and sepsis, to name a few [18]. However, despite an exhaustive list of potential causes, muscular atrophy is often attributed to an imbalance between the catabolic and anabolic signaling pathways [19]. Thus, with metabolic problems, changing lifestyles, and ageing, muscular atrophy poses a bigger health concern than ever before [20].

Conditions like sarcopenia that forms the quintessence of most MSDs is characterized by a reduction of muscle mass protein, which eventually culminates into a loss of muscle function [21]. It is often marked by a steady and extensive diminution of skeletal muscle potency, which often climaxes with physical impairment and muscle immobility [22]. It is often conspicuous in the elderly cohorts, as it is strongly corroborated by frailty development [21].

The current understanding of muscle damage and pain couples muscle damage and associated pain with the generation of reactive oxygen species (ROS), oxidative stress, and inflammation [23]. Mounting bodies of evidence have substantiated the utility of several plants, plant extracts, and phytochemicals as pharmacotherapeutic interventions to scavenge ROS and ameliorate inflammation, thereby intercepting the pathogenesis of these MSDs.

## 3. Signalling Pathways and Skeletal Muscle Homeostasis

In response to various environmental stresses, the skeletal muscles trigger a spectrum of signaling pathways that facilitate the latter to remodel and maintain muscular action. One of the quintessential pathways that are necessary for self-renewal and differentiation of adult muscle cells is the Wnt scheme. Recent studies have also elucidated its importance not only in the proliferation of adult stem cells but also in the improvement of embryonic muscle cells [24]. Wnt signaling is an enduring and evolutionarily conserved system proven to be essential for adipose tissue formation. This path exerts its influence on a variety of biological processes, including muscle tissue development and the creation of the motor neuron-muscle synapse. Wnt signaling influences myogenesis by stimulating differentiation at early developmental stages. However, interception of this signaling would result in poor and deformed skeletal muscle formation. Wnt signaling also regulates the location of several proteins vital for synapse formation as well as precise muscle contraction throughout the maturation of the neuromuscular junction (NMJ). This signaling also plays a profound role in muscle fibrosis because it interacts with other profibrotic molecules such as connective tissue growth factor (CTGF) and transformative growth factor-*β* (TGF-*β*) [25].

Another key signaling effector involved in skeletal muscle development is the fibroblast growth factor (FGF) and the ubiquitin-proteasome pathway. The loss of FGFR1 signaling results in decreased skeletal mass, thereby disrupting the myofiber structure [26]. While some interventions have a favorable impact on the development of skeletal muscles, others may have an antagonistic effect. The induction of muscular atrophy is an interplay of various transcriptional mechanisms, which catalyze the degradation as well as the production of new proteins from the old ones [27, 28]. In addition, the expression of MurF1 and MAFbx/Atrogin-1 is considerably enhanced in the atrophies of skeletal muscles [29–32]. The FoxO transcription factor family, which is phosphorylated by Akt, is involved in the suppression of MuRF1 and MAFbx/Atrogin-1 [32, 33]. FoxO transcription factors, such as FoxO1 and FoxO3, translocate to the nucleus after being dephosphorylated and upregulating MurF1 and MAFbx/Atrogin-1 [32, 33].

In addition, Insulin-like Growth factor (IGF-1) has a profound effect on myoblast differentiation and proliferation of skeletal muscles. IGF-1 also stimulates the phosphatidylinositol-3 kinase (PI3K)/Akt and other downstream targets like mTOR, that facilitate the upregulation of protein synthesis, further resulting in the hypertrophy of skeletal muscle cells [34, 35].

**Figure 1:**
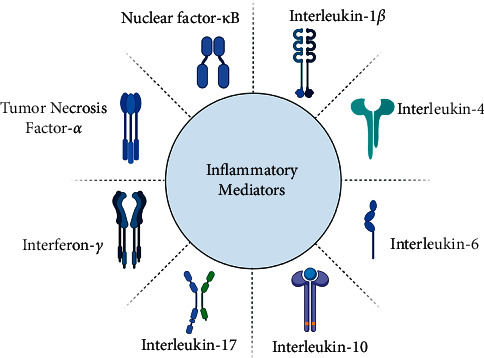
Inflammatory mediators involved in the pathophysiology of musculoskeletal diseases.

Another signaling pathway involved in atrophy of the skeletal muscles is nuclear factor-kappa B (NF-*к*B). In the cultured C2C12 myoblasts without NF-*к*B, the addition of TNF-*α* mediated the suppression of muscle development [36]. Similarly, through the regulation of MuRF1, the overexpression of NF-*к*B endorsed an acute atrophy of the skeletal muscles. Upon injury, the adult skeletal muscle triggers a variety of important inflammatory mediators, which aid in controlling the healing environment to promote faster healing. However, some of the inflammatory cytokines have been attributed to both physiological as well as pathological activities [37–39]. These cytokines have been enlisted in Figure 1.

## 4. Phytomedicine in the Treatment of MSDs

**Figure 2:**
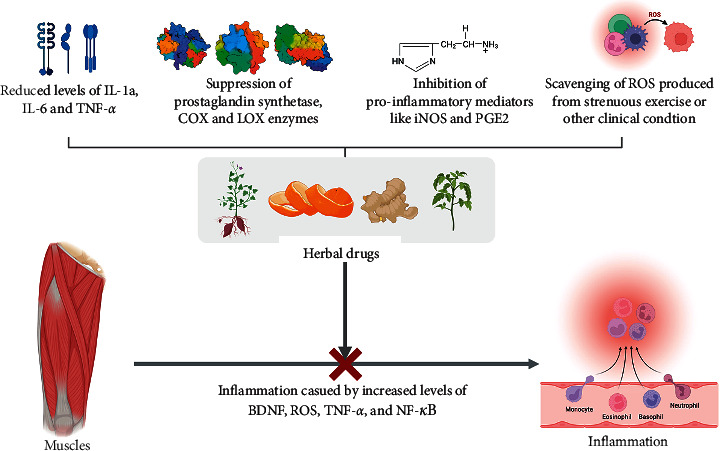
Mechanism of medicinal herbs and phytochemicals in the inhibition of skeletal muscle inflammation. (BDNF = brain-derived neurotropic factor; COX = cyclooxygenase; IL = interleukin; iNOS = inducible nitric oxide synthase; LOX = lipoxygenase; NF-*к*B = nuclear factor-*к*B; PGE2 = prostaglandin E2; ROS = reactive oxygen species; TNF-*α* = tumor necrosis factor-*α*).

Since antiquity, plants have been a rich source of pharmacologically active phytochemicals and therapeutic moieties. They have been used as a safe and efficient supplementary therapy for a plethora of disease processes [40–42]. These herbs not only increase the blood circulation but also repair the damaged skeletal tissue, thereby alleviating skeletal muscle inflammation and MSDs [7]. Over the years, several botanicals and medicinal herbs have been found to positively affect the treatment of skeletal muscle problems, including muscle thinning and muscle atrophy, thereby improving the treatment outcome [43]. The production of ROS, followed by inflammatory cascade and oxidative stress, often precedes the impairment of the muscle tissue and causes muscle ache [12]. The rationale behind the administration of herbal drugs as a curative for skeletal muscle inflammation among most MSDs is elucidated in Figure 2.

## 5. Role of Phytotherapy in the Management of Skeletal Inflammation in MSDs

With a paradigm shift in the dietary preferences, nature of work, and lifestyles of individuals, the prevalence of skeletal muscle inflammation and MSDs is on the rise. With this increment in MSDs, there is a pressing need for dietary management to alleviate the symptoms associated with the former. Nonetheless, the prevention strategy and medication regimen would largely be specific to the MSD, taking into account the rate of progression, comorbidities, and characteristics of the individual. However, the consumption of medicinal plants could potentially prevent muscle wasting and supplement the muscles with essential nutrients, while various bioactives could intercept the signaling pathways that progress the disease. Some of the prominent medicinal herbs employed for the treatment of the latter have been elucidated below.

### 5.1. *Coffea arabica*

The *Coffea arabica,* or coffee plant, is a subset of the family *Rubiaceae* and genus *Coffea*. It is generally a woody perennial tree that thrives at higher altitudes. Although caffeine is the major component of *C. arabica*, it also contains dimethylxanthines, paraxanthine, theobromine, and theophylline, which are metabolized in the liver by CYP1A2 enzymes [44]. These phytochemicals demonstrate a spectrum of activities, ranging from boosting sensations to enhancing focus [45]. Other trace compounds present in coffee include polyphenols such as hydroxyhydroquinone (HHQ) and chlorogenic acids, among others [44].

The administration of *C. arabica* extract has been associated with a plummeting in levels of interleukins IL-1a, IL-6, and TNF-*α*, which have been attributed to grip strength and muscle mass. The results from an *in-vitro* murine model demonstrated an increment in the count of proliferating tissues and enhanced nucleic acid synthesis via the Akt signaling pathway, after administration of *C. arabica* extract. The latter also increased the activation of satellite cells while concurrently lowering inflammatory cytokines, thereby validating its antiinflammatory properties. These immunomodulatory properties and antioxidant potential are attributed to compounds like kahweol [46].

### 5.2. *Zingiber officinale*

Ginger (*Zingiber officinale*) is a subset of the *Zingiberaceae* family and is predominantly found in traditional medicine systems. It is generally cultivated in southeast Asia and then exported globally, to be employed both as a spice and a condiment [47]. The utility of ginger both as a dietary supplement and an herbal medicine is attributed to its rich phytochemistry (reviewed in [48]). Owing to its potential antioxidative and antiinflammatory properties, it finds its utility in antiageing and degenerative disorders such as arthritis and rheumatism. Additionally, its antimicrobial effect also helps in the treatment of sepsis-induced MSDs [48–52]. A recent study reported a moderate reduction in muscular discomfort progression for one to two days, when they consumed ginger 24 hours after their exercise session. However, the consumption of heat-triggered ginger did not potentiate this activity [53]. The gingerol, shogaol, and other chemical moieties present in ginger have been studied to inhibit prostaglandin and leukotriene biosynthesis via suppression of the prostaglandin synthetase and 5-lipoxygenase enzymes (reviewed in [52]). In addition, they also inhibit the synthesis of other pro-inflammatory cytokines, including IL-1, IL-8, and TNF-*α* [54, 55]. A study conducted by Pan et al. concluded that shogaol suppresses the expression of iNOS and COX-2 genes in macrophages [56]. In addition, Jung et al. demonstrated the inhibition of excessive NO, PGE (2), TNF-*α*, and IL-1*β* by the rhizome hexane fraction of ginger [57]. A study by Habib et al. showed that the extract of *Z. officinale* normalised the expression of TNF-*α* and NF-*к*B in liver cancer-induced murine models [58]. However, the study by Lantz et al. exhibited the inhibitory effect of LPS-induced COX-2 expression while shogaol-containing extracts had no effect on COX-2 expression [59]. These studies substantiate the utility of ginger extracts as a potential antiinflammatory and antioxidant [52]. However, the studies that evaluated the efficacy of ginger in osteoarthritis (OA) patients showed conflicting results. While one study proved its effectiveness to be statistically significant in alleviating the symptoms associated with knee OA [60], the other opined its utility to be confined only to the first half of the treatment regimen [61]. However, recent evidence validates the efficacy of [6] shogaol in gouty arthritis, owing to its potential antioxidant and antiinflammatory activity [62]. These effects render ginger and its extract as a potential treatment modality in alleviating the inflammation associated with MSDs.

### 5.3. *Curcuma longa*

Turmeric is a spice that has garnered attention from both the medical as well as the culinary communities [63–65]. Turmeric (*Curcuma longa*) is a rhizomatous herbaceous perennial plant and a subset of the *Zingiberaceae* family [66]. Despite the knowledge of its therapeutic benefits for ages, its exact mechanism of action has only been elucidated in the recent years [67]. The chemical compound predominantly found in *C. longa* is curcumin (4) or diferuloylmethane, which is attributed to its antioxidant, antibacterial, antimicrobial, antimutagenic, antiinflammatory, and anticancer properties [67–73]. Its utility has been demonstrated in the alleviation of pain [74], metabolic syndrome [75], inflammatory and degenerative conditions [67, 76, 77]. Recent studies have underscored the positive effect of curcumin on muscles by reducing the activation of the NF-*к*B signaling pathway. This is imperative for the alleviation of the delayed onset muscle syndrome (DOMS), as the NF-*к*B pathway is involved in the control of proteolysis and muscle inflammation, thereby exerting a muscle-protective effect. The administration of curcumin has not only been attributed to the reduction of muscle loss following sepsis and endotoxemia but also to inducing the regeneration of the wasted muscle after the traumatic injury. Curcumin exerts its antiinflammatory and antioxidant properties via the inhibition of NF-*к*B signaling, induction of heat shock response, abrogating the p38 kinase activity, inhibition of oxygen free radical formation, and prevention of the biosynthesis and release of pro-inflammatory cytokines (reviewed in [78]). Furthermore, curcumin reduces the inflammation associated with exertive exercise-induced muscle damage. This is achieved by the neutralization of pro-inflammatory cytokines and muscle injury markers such as creatine kinase (Ck) [79]. It also increases the expression of glucose-regulated protein 94 kDa (Grp94) in myogenic cells, which often plummets in unloaded muscles but is essential in the prevention of myofiber atrophy [80, 81].

### 5.4. *Aloe barbadensis*

*Aloe vera*, or *Aloe barbadensis*, is a tropical, drought-resistant succulent plant, belonging to the family *Liliaceae*. It is native to the Mediterranean region, India, China, and eastern Africa (reviewed in [82]). Aloes contain a copious amount of bioactive compounds, such as anthraquinones (7), fatty acids, flavonoids, lectins, terpenoids, monosaccharides, and polysaccharides (glucomannan, hemicelluloses, and pectins), tannins, sterols (campesterol and *β*-sitosterol), enzymes, salicylic acid, minerals (Fe, Cr, Cu, Ca, Mg, Na, K, P, Zn, and Mn), and vitamins (including Vit-A, Vit-C, Vit-E, *β*-carotene, Vit-B1, Vit-B2, Vit-B3, Vit-B6, choline, Vit-B12, and folic acid) (reviewed in [83–88]). The abundance of these compounds in *A. barbadensis* accounts for its potent antiinflammatory and antiarthritic effects (reviewed in [89]). Despite its utility in a spectrum of fields, *A. barbadensis* is very effective in the treatment of allergic reactions, arthritis, and rheumatoid fever. Growing bodies of evidence attribute this antiinflammatory property to the bioactive compound aloe-emodin, an anthraquinone alkaloid that exerts its effect by inhibiting prostaglandin-E2 and inducible nitric oxide [90, 91]. These properties could be extrapolated for the treatment of inflammation associated with MSDs.

### 5.5. *Citrus limon*

*Citrus limon,* or lemon, is a subset of the family *Rutaceae*. It is a tree with evergreen leaves and yellow edible fruits, which upon processing yields the juice and essential oil (reviewed in [92, 93]). The pharmacologically active phytoconstituents of *C. limon* juice extract and essential oils include flavones (apigenin (9), diosmin, orientin, and vitexin); flavanones (eriodyctiol, hesperidin (1), hesperitin, naringin, and neohesperidin); flavanols (quercetin (8), limocitrin, and spinacetin) and their derivatives. Other trace quantities in *C. limon* include eriocitrin, limonin, and nomilin (reviewed in [94]). The *C. limon* leaf extract is also known to scavenge the free radical levels and inhibit the redox reaction of xanthine, thereby proving as a potent anti-oxidant [95]. Concurrently, there is also a decrease in the levels of pro-inflammatory molecules such as ROS and prostaglandin scaffolds. Thus, owing to its potential antiinflammatory actions, several preparations of this plant can alleviate joint inflammation and arthritis [96].

### 5.6. *Glycyrrhiza glabra*

Liquorice (*Glycyrrhiza glabra*) is a plant belonging to the *Leguminosae* family. It is grown in China, India, Italy, Iran, Russia, and Spain [97]. A spectrum of components has been isolated from the roots of *G. glabra*, which was made up of simple sugars, triterpenes, saponins, flavonoids, polysaccharides, pectins, amino acids, mineral salts, asparagines, bitters, essential oil, fat, estrogen, gums, mucilage (rhizome), proteins, resins, starches, sterols, volatile oils, tannins, glycosides, among other substances [98–100]. The flavonoid component of *G. glabra* contains liquiritin, isoliquiritin (a chalcone), and other chemicals that give its characteristic yellow hue [101]. In addition, isoflavones like glabridin and hispaglabridins A and B have substantial antioxidant action [102], while both glabridin and glabrene exhibit estrogen-like activity [103]. Estradiol has been proven to exert a beneficial effect on skeletal muscles by stimulating the proliferation of satellite cells. Owing to the presence of estradiol-specific receptors on muscle fibers, skeletal muscles tend to respond to differences in estrogenic hormones. Furthermore, estradiol potentially limits and mitigates the stress damage imparted on the skeletal muscle [104]. Recent studies have attributed the antiinflammatory properties of liquorice to its inhibitory effect on the production of IL-6, NO, PGE2, and TNF-*α* [105]. Furthermore, the hydromethanolic liquorice root extract demonstrated potent antioxidant activity in an *in-vitro* system [106]. Additionally, licochalcones B and D have the ability to substantially reduce microsomal lipid peroxidation [97]. Moreover, retrochalcones show mitochondrial lipid peroxidation and inhibit oxidative hemolysis in red blood corpuscles (RBCs). Additionally, compounds like glabridin, hispaglabridin A, 3′hydroxy-4-O-methylglabridin, and dehydrostilbene derivatives such as dihydro-3,5,4-trihydroxy-4,5-diiodopentenylstilbene have also been reported for their potential antioxidant capacities [97, 107–109]. Growing bodies of evidence have validated the antiinflammatory effect of glycyrrhizin to be comparable to that of hydrocortisone and other corticosteroid hormones upon being broken down in the gut [97].

### 5.7. *Citrus aurantium*

The *Citrus aurantium*, popularly known as bitter orange is a subset of the family *Rutaceae* [110, 111]. It is mainly employed as a flavoring and acidifying agent in the food industry [112, 113]. The plant is rich in essential oils, vitamins, minerals, phenolic compounds, terpenoids, and flavonoid-type compounds that demonstrate a spectrum of biological effects, ranging from anxiety and obesity to lung and prostate cancers [110, 112, 114–119]. Among the diverse phytoconstituents present in *C. aurantium*, the pharmacological activity is attributed to the flavonoids present in them [110, 120–122]. The flavonoids (such as hesperidin, nobiletin (2), and naringin (3)) in *C. aurantium* prevent the inflammatory sensation in L6 skeletal muscle cells that is propagated by the lipopolysaccharides (LPS). Furthermore, the flavonoids isolated from Korean *C. aurantium* potentially inhibit the inducible nitric oxide synthase (iNOS), cyclooxygenase-2 (COX-2), IL-6, and TNF-*α* by intercepting the mitogen-activated protein kinases (MAPKs) and NF-*к*B signaling pathways. Moreover, these flavonoids demonstrated antiinflammatory properties by modulating the proteins involved in the immunological response. Furthermore, pretreatment with the flavonoids substantially reduced the quantity of cleaved caspase-3, a protein generated during muscle inflammation and highlighted in muscle proteolysis and atrophy [123]. These studies validate its utility in reducing the inflammation associated with MSDs.

### 5.8. *Alstonia scholaris*

Saptaparni or *Alstonia scholaris*, belonging to the family *Apocynaceae*, has been an integral part of the ethnopharmacological armamentarium, especially in the “dai” ethnopharmacy in the treatment of respiratory ailments [124]. This utility has reinforced its potential in other inflammatory ailments. The comprehensive investigation of various plant parts of *A. scholaris* reported a spectrum of indole alkaloids, iridoids, monoterpenoids, and terpenoids [124–133]. It is also rich in alkaloids (like echitamine, tubotaiwine, akuammicine, echitamidine, picrinine, and strictamine) and terpenes, which are used as antiinflammatory agents [134, 135]. The ethanolic extract of *A. scholaris* leaves in a dose of 100–200 mg/kg demonstrated a significant reduction in the total leukocyte migration, while concurrently reducing the levels of pro-inflammatory mediators like COX, LOX, PGE2, and NO in animal models [136]. The plant has also afforded protection in various pain and inflammation models, including the formalin test, acetic acid-induced writhing, and the air pouch model in rodents [124]. A study by Subraya and Gupta concluded the significant antiinflammatory effect of the methanolic extract of *A. scholaris* stem bark compared to indomethacin against carrageenan-induced acute pedal edema, cotton pellet-induced, and dextran-induced edema [137–139]. The dextran-mediated inflammation was thought to be alleviated by the antihistaminic property of the extract. However, the antiinflammatory effect of the cotton pellet granuloma test reflected its efficacy in inhibiting the proliferation of fibroblasts and synthesis of collagen and mucopolysaccharide during the formation of granuloma tissues [137, 138]. Despite the various studies underscoring its utility as an antiinflammatory agent, substantial bodies of evidence are required to substantiate its efficacy in the treatment of MSDs.

### 5.9. *Eysenhardtia polystachya*

*E. polystachya* is a subset of the family *Fabaceae* and is predominantly found throughout Mexico and in the southeastern United States [140, 141]. The various parts of the tree is copious in a variety of secondary metabolites, including chalcones, coumarins, dihydrochalcones, fatty acids, flavonoids, flavones, flavanones, isoflavonoids, phenols, pterocarpan, and sugars to name a few [141]. These active congeners have antiinflammatory properties and have been used in the treatment of arthritis [142–144]. A recent study explored the antiinflammatory potential of *E. polystachya* bark in its hexane, chloroform, and methanolic extracts on Wistar rat models. These studies were also supplemented with the carrageenan and croton oil-induced edema tests, following which the PGE2, IL-1*β*, TNF-*α*, and leukotriene B4 (LTB4) levels were quantified. Furthermore, the methanolic extract of *E. polystachya* bark showed antiinflammatory activity in the different models by inhibiting the expression of cytokines like PGE2, IL-1*β*, TNF-*α*, LTB4, and the enzymes lipoxygenase and xanthine-oxidase linked to the inflammatory problems [141, 145]. In addition, the researchers elucidated the effects of an ethanolic extract from the bark of E. polystachya on Complete Freund's Adjuvant (CFA)-induced rheumatoid arthritis. The secondary metabolites of *E. polystachya*, mainly flavonoids, inhibited the secondary inflammation in arthritic rats, promoting the histopathological alterations while concurrently lowering the levels of circulating pro-inflammatory cytokines [143]. Moreover, the study on the antiinflammatory effect of the leaves and branches of *E. polystachya* used *in-vitro* tests that stimulated the macrophages using LPS. These results validated a reduction in H_2_O_2_ (IC50 = 43.9 ± 3.8 *μ*g/mL) and IL-6 (73.3 ± 6.9 *μ*g/mL) [142]. These studies corroborate the utility of this plant in treating inflammation and could be extrapolated for alleviating the inflammation associated with MSDs.

### 5.10. *Ipomoea batatas*

The sweet potato, or *I. batatas,* is a plant often associated with being a major crop food worldwide. It is widely produced and consumed in East Asia, Oceania, and sub-Saharan Africa, with China being the highest producer, with 76.07% of the world's production [146–148]. The leaves of *I. batatas* are widely regarded as a functional food that poses a variety of health-promoting benefits [146, 149]. *I. batatas* is a subset of the *Convolvulaceae* family and is a rich source of antiinflammatory phytonutrients such as rutin, gallic acid, quercetin, and kaempferol (5). The flowers of the latter show their antiinflammatory effect by modulating the levels of IL-1b, IL-6, and NO [150]. The latter is rich in Vit B1, Vit B5, Vit B6, niacin, riboflavin, polyphenols (anthocyanin), phenolic compounds (including caffeic, monocaffeoylquinic, dicaffeoylquinic, and tricaffeoylquinic acids), triterpenes (such as *β*-carotene and boehmeryl acetate), trace elements (calcium, iron, and zinc), and other proteins [151–155]. The leaves and areal parts of *I. batatas* contain more polyphenols than most vegetables, with atleast 15 anthocyanins and 6 polyphenolic compounds [151]. The dry powder of *I. batatas* tuber and roots were extracted using ethyl acetate and methanol. Various tests, including carrageenan-induced paw edema test, croton oil-induced ear, and anal edema inhibition, and CFA-induced antiarthritic experiments, were performed on Sprague-Dawley rats at a dose of 300 mg/kg body weight. Their antiinflammatory potential was attributed to the suppression of pro-inflammatory cytokines (such as PGE2, IL-1*β,* and TNF-*α*), with a concurrent reduction in NO levels, in the ethyl acetate root extract and methanolic tuber extract, respectively [150]. These studies validate their efficacy in ameliorating inflammation in most tissues.

### 5.11. *Swertia chirayita*

*S. chirayita* is a popular herbal plant that has been used for ages [156, 157]. The plant mainly matures at tall altitudes of 1200–1500 metres in sub-temperature regions, specifically on the slopes of the Himalayas, ranging from Bhutan to Kashmir [158] (reviewed in [159]). The medicinal plant has been employed for a multitude of clinical applications, including vomiting, hepatitis, constipation, inflammation, weak stomach, and indigestion [157, 160, 161]. Growing bodies of evidence have analyzed the hepatoprotective activity of this plant extract to be associated with its antioxidant property [157]. An oral dose (200 mg/kg) of *S. chirayita* ethanolic leave extract decreases pro-inflammatory cytokines such as TNF-*α* and IL-1*α,* as well as paw edema in arthritic rats [162]. Chen et al. used many common *in-vitro* methods to investigate the antioxidant potential of *S. chirayita* plant extract against CCl4-induced toxicity in murine models. The characteristics of malondialdehyde (MDA) and antioxidant enzymes such as glutathione (GSH), superoxide dismutase (SOD), and catalase (CAT) have also been elucidated using standard investigation techniques. Furthermore, the powder obtained by evaporating and lyophilizing an ethanolic extract of *S. chirayita* was found to have strong antioxidant properties [161]. A study by Das and Barman explored the analgesic and antiinflammatory properties of the ethanolic plant root extract in the carrageenan-induced rat paw edema model. In order to determine the analgesic property of the extract, a writhing test based on acetic acid induction and radiant heat tail-flick methods were used. At a 400 mg/kg dose, the extract was efficient in reducing the development of edema, resulting in a 57.81% reduction in edema volume, 3 hours after administration. This study substantiates its utility in having analgesic and antiinflammatory activities [163]. Banerjee et al. tested a xanthone derivative for inflammation from *S. chirayita* in acute, sub-acute, and chronic male rats. Their research concluded its antiinflammatory activity to be comparable to that of diclofenac [164]. Therefore, these studies underscore its utility in inflammation and could be a potential treatment modality in inflammation in MSDs.

### 5.12. *Strychnos nux-vomica*

The strychnine tree, or *Strychnos nux-vomica,* belongs to the *Loganiaceae* family and has demonstrated remarkable treatment potential for several disorders. They have been employed for a variety of therapeutic outcomes, including analgesic, antipyretic, cytotoxic, and antiinflammatory activities. These phytotherapeutic effects have been attributed to the compounds present in them, like 7-hydroxy coumarin, Kaempferol-3-rutinoside, Kaempferol-7-glucoside, Quercetin-3-rhamnoside, and rutin. A study by Eldahshan and Daim validated the antiinflammatory, antipyretic, and antinociceptive effects of *S. nux-vomica* leaf extract in animal models. These activities were attributed to the inhibitory effect of the extract and its phytoconstituents on inflammatory mediators such as PGE2 and TNF-*α* [165]. In addition, the *S. nux-vomica* extract demonstrated a reduction in PGE2, with a concurrent decrease in vascular permeability, thereby demonstrating potent antiarthritic and antiinflammatory activities, which are profound in the case of joint inflammation [166–168]. However, for preclinical applications, the effects and potential toxicities must be elucidated through further tests.

### 5.13. *Rosemarinus officinalis*

Rosemary, or *Rosmarinus officinalis,* is a medicinal plant, based out of the Mediterranean region, but eventually cultivated across the globe. Besides its potential therapeutic utility, it is commonly employed as a condiment and a food preservative. By virtue of its phytoconstituents, the plant demonstrates potential pharmacological activities, including antioxidant, antiinflammatory, antimicrobial, antitumour, antiproliferative, protective, inhibitory, and attenuating effects [169–173]. The phytoconstituents present in *R. officinalis* mainly include *α*-pinene, caffeic acid (10), camphor, carnosic acid, carnosol, chlorogenic acid, eucalyptol, monomeric acid, oleanolic acid, rosmarinic acid, rosmadial, rosmanol, rosmaquinones A and B, secohinokio, ursolic acid, and the derivatives of eugenol (6), and luteolin [173–178]. The effects of *R. officinalis* were evaluated in patients with osteoarthritis, rheumatoid arthritis, and fibromyalgia for four weeks in an open-label trial; the hs-CRP levels that mark the presence of inflammation had also reduced substantially in patients who had demonstrated the augmentation in this index. Though the reduction in inflammation was related to the pain score, no remission was observed in fibromyalgia scores [179]. Recent shreds of evidence corroborate the antiinflammatory effect of *R. officinalis*. This effect is attributed to the disruptive effect of rosmarinic acid on the activation of the complement system by reducing the C3b attachment, while the dose required for the latter is relatively low (34 *μ*M) [180]. This plant has also been demonstrated to have topical antiinflammatory effects in murine models [181]. These effects validate its efficacy as a potential antioxidant and antiinflammatory agent.

### 5.14. *Borago officinalis*

The borage, or *Borago officinalis,* is a member of the *Boraginaceae* family and is native to the European area as well as the north of Africa [182]. Since antiquity, borage and its derivatives have formed a part of the traditional medicine armamentarium, especially in the treatment of allergic disorders, owing to their immunomodulatory properties [183]. The plant is a rich source of gamma linoleic acid (GLA), containing about 25% of GLA. The GLA augments the cyclic adenosine monophosphate (cAMP) by elevating the prostaglandin-E (PGE) levels, as well as being a strong suppressor of TNF-*α* [184]. Its activity is associated with its ability to suppress the TNF-*α,* while delivering gamma-linolenic acid [183]. This mechanism explains its antiinflammatory effect in the alleviation of symptoms associated with rheumatoid arthritis. However, this treatment modality has been contraindicated as an intervention in pregnant women, as it is associated with an increased risk of miscarriage [184]. The antirheumatoid arthritis potential of borage seed oil of 1.4 gm/day when compared to the placebo group exhibited 36.8% amelioration post 6 months of therapy. Additionally, a 2.8 gm/day administration of borage seed oil in the treatment of rheumatoid arthritis demonstrated a 64% amelioration in the treatment group compared to the 21% in the control group post 6 months of therapy (reviewed in [185, 186]). These clinical studies underscore their utility as a potential antirheumatoid arthritis and antiinflammatory medication.

### 5.15. *Harpagophytum procumbens*

The Devil's claw, or *Harpagophytum procumbens,* is a member of the *Pedaliaceae* family [187]. The pharmacological activity of its extract is mainly associated with its copiousness of an antiinflammatory compound, Harpagoside [188]. The Devil's claw root extract has been claimed to inhibit NO levels and other pro-inflammatory cytokines (including PGE2, IL-6, IL-1*β*, and TNF-*α*). It also prevents the metabolism of arachidonic acid and the biosynthesis of eicosanoids, which further inhibits the COX-2 enzyme, thereby reducing inflammation [189–191]. A pilot study has been carried out on patients suffering from rheumatoid arthritis and psoriatic arthritis. The patients were administered 410 mg of the devil's claw liquid extract TDS. However, the patients did not show any remission or subjective and objective improvement in their clinical condition [192]. Additionally, another preclinical study involving devil's claw showed no efficacy in improvement in the carrageenan-induced edema in the hind foot of the rat [193]. These studies have described the potential of devil's claw extract in inflammatory MSDs including rheumatoid arthritis and psoriatic arthritis. However, further studies need to be carried out to substantiate their utility and toxicity profiles.

**Figure 3:**
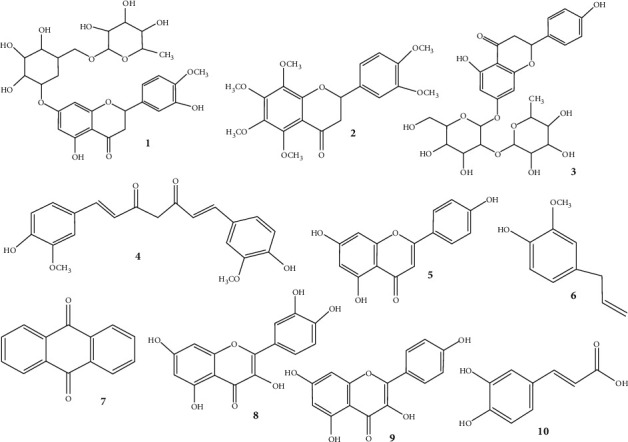
Various phytochemicals that exert their effects on inflammation in musculoskeletal disorders.

**Figure 4:**
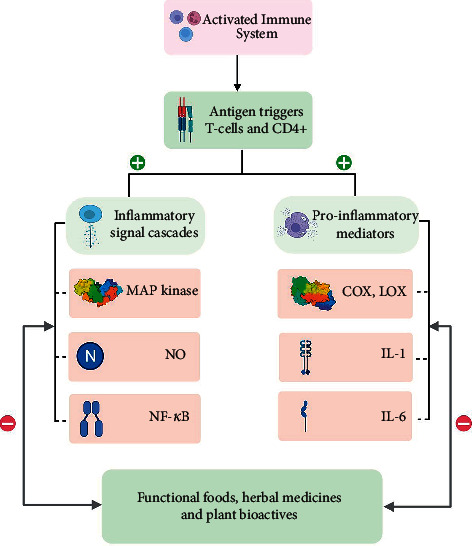
General protective actions mediated by functional food, herbal medicines, and their derived phytoconstituents.

Various phytochemicals effective in inflammatory pathophysiology involved in musculoskeletal disorders have been elucidated in Figure 3, while their protective actions have been demonstrated in Figure 4. Further investigations are warranted to understand the clinical efficacy of these bioactive phytomedicines along with the exploration of new potential botanicals. Safety studies play a crucial role in the drug development process [194, 195], therefore they should also be carried out. The research summary has been concisely presented in Table 1. Additionally, the recent clinical advances have been curated and tabulated in Table 2.

## 6. Conclusion and Outlook

Although the utility of various medicinal plants and their derived phytochemicals has been established in the treatment of various musculoskeletal disorders, especially rheumatoid and psoriatic arthritis, however, their ethnopharmacological studies and evidences presented are not convincing, and thorough evidence needs to be provided to substantiate their utility, toxicity profiles, dosing, and formulation in the mitigation and management of inflammation in the MSDs. Additionally, it aims to review the various plants that have been long lost as a potential modality in alleviating the inflammation and pain associated with various MSDs and their role as a part of the diet or as a medicinal treatment intervention. The current literature available is limited and more detailed *in vitro* and *in vivo* studies are required to provide a better view of the current treatment landscape of inflammation and related diseases of the musculoskeletal system. However, the authors also opine on the need for further preclinical and clinical studies to corroborate the effects in humans. In addition, these phytochemicals could be extracted to potentially fabricate them into a viable formulation, for clinical administration. Moreover, more cogent and exhaustive data is required for understanding the molecular and signaling mechanisms and the way in which these crude drugs and/or phytoconstituents must be administered and their potential adverse effects and toxicity must be catered after consumption.

## Figures and Tables

**Table 1:** Plant bioactives in the management of musculoskeletal disorders.

Dietary food or medicinal herb	Family	Phytochemical molecule	Research summary	References
*Coffea arabica*	*Rubiaceae*	Caffeine dimethylxanthines, paraxanthine, theobromine, theophylline	↓ in IL-1a, IL-6, and TNF-*α*	[46]
			↑ proliferation of the tissues	
			↑ in the nucleic acid synthesis via the Akt signaling pathway	
			↓ in the levels of circulating inflammatory cytokines	
			↑ activatin of satellite cells	

*Zingiber officinale*	*Zingiberaceae*	Gingerol, shogaol	↓ in the production of prostaglandins and leukotrienes, via the suppression of the prostaglandin synthetase 5-lipoxygenase enzymes	[51, 52, 54–57]
			↓ the IL-1, IL-8, and TNF-*α* synthesis	
			↓ the expression of iNOS and COX-2 genes	
			Hexane fraction of ginger extract ↓the excessive NO, PGE2, TNF-*α,* and IL-1*β*	

*Curcuma longa*	*Zingiberaceae*	Curcumin or diferuloylmethane	↓ the activation of the NF-*к*B signaling pathway	[78, 80, 81]
			↑ expression of glucose-regulated protein 94 kDa (Grp94) in myogenic cells	

*Aloe vera* or *Aloe barbadensis*	*Liliaceae*	Campesterol, *β*-sitosterol	↑ the inhibition and ↓ the expression of PGE2 and inducible nitric oxide.	[90, 91]

*Citrus limon*	*Rutaceae*	Quercetin, limocitrin, and spinacetin	↓ the pro-inflammatory molecules such as ROS, PG scaffolds, and precursors	[95, 96]
			↓ free radical levels, by means of scavenging, in its leaf extract	
			Inhibition of xanthine redox reaction	

*Glycyrrhiza glabra*	*Leguminosae*	Hispaglabridins A and B	↓ the synthesis of IL-6, NO, PGE2, and TNF-*α*	[97, 106–109]
			↓ in the microsomal lipid peroxidation	
			Potent antioxidant effect in its hydromethanolic extract	

*Citrus aurantium*	*Rutacea*e	Essential oils, vitamins, minerals, phenolic compounds, terpenoids	Inhibition of iNOS, COX-2, IL-6, and TNF-*α*, by inhibiting the MAPKs and NF-*к*B signaling pathways	[123]

*Alstonia scholaris*	Apocynaceae	Echitamine, tubotaiwine, akuammicine, echitamidine, picrinine and strictamine	↓in total leukocyte migration	[136]
			↓ in the biosynthesis of pro-inflammatory mediators such as COX, LOX, PGE2, and NO, upon validation of its ethanolic extract	

*Eysenhardtia polystachya*	*Fabaceae*	Coumarins, dihydrochalcones, fatty Acids, flavonoids	↓ in the expression of PGE2, IL-1*β*, TNF-*α*, LTB4, in a methanolic extract	[140, 141]
			↓ in the expression of enzymes LOX and xanthine-oxidase when a methanolic extract was used	

*Ipomoea batatas*	*Convolvulaceae*	Vit B1, vit B5, vit B6, niacin, riboflavin, polyphenols	Modulation of IL-1*β*, IL-6, and NO	[150]
			↓ in the activity of PGE2, IL-1*β*, and TNF-*α*, upon methanolic tuber extraction	
			↓ in NO levels, upon administration of ethyl acetate root extract	

*Swertia chirayita*	*Gentianaceae*	Chiratin, xanthone derivative	Ethanolic leaves extract ↓ the activity of TNF-*α* and IL-1*α*	[162]

*Strychnos nux-vomica*	*Loganiaceae*	Quercetin-3-rhamnoside, and rutin	Leaves extract ↓ the effect exerted by PGE2 and TNF-*α*	[165–168]
			Antiarthritic and antiinflammatory effect attributed to the ↓ of PGE2 levels	

*Rosemarinus officinalis*	*Lamiaceae*	Rosmarinic acid, rosmadial, rosmanol, rosmaquinones	Rosmarinic acid disrupts the activation of complement system	[180]
			↓ in the C3b attachment at low doses	
*Borago officinalis*	*Boraginaceae*	Gamma-linolenic acid (GLA),	↓ in the expression and activity of TNF-*α*	[183, 184]

*Harpagophytum procumbens*	*Pedaliaceae*	Harpagoside	↓ in the total levels of NO, PGE2, IL-6, IL-1*β*, and TNF-*α*	[189–191]
			Intercepts the metabolism of arachidonic acid and the synthesis of eicosanoids, thereby inhibiting COX-2 enzyme	

↓ represents a decreasing trend and ↑ represents an increasing trend.

**Table 2:** Clinical trials testing the efficacy of the medicinal herbs and plant bioactives in the treatment of musculoskeletal inflammation.

Sr. no.	Title	Status	Conditions	Interventions	Characteristics	NCT number
1.	Aloe vera ointment application and skeletal muscle recovery	Completed	(i) Skeletal muscle damage (ii) Exercise-induced aseptic inflammation (iii) Performance	(i) Biological: placebo (ii) Biological: natural aloe vera (iii) Biological: aloe vera soup	Study type: interventional Phase: not applicable Study design: (i) Allocation: randomized (ii) Intervention model: crossover assignment (iii) Masking: double (participant, investigator) (iv) Primary purpose: treatment Outcome measures: (v) Change in creatine kinase activity in plasma (vi) Change in performance of knee extensor muscles (vii) Change in delayed onset of muscle soreness	NCT03934762

2.	Describing Chinese herbal medicine telehealth care for symptoms related to infectious diseases such as COVID-19	Recruiting	(i) Coronavirus infection	(i) Dietary supplement: Chinese herbal medicine	Study type: observational Phase: study design: (i) Observational model: ecologic or community (ii) Time perspective: prospective Outcome measures: (iii) Patient reported main complaint (iv) Conduct qualitative analyses of data	NCT04380870

3.	PB125, osteoarthritis, pain, mobility, and energetics	Recruiting	(i) Osteoarthritis, knee (ii) Muscle weakness (iii) Pain, joint	(i) Dietary supplement: PB125 (ii) Dietary supplement: placebo	Study type: interventional Phase: not applicable Study design: (i) Allocation: nonrandomized (ii) Intervention model: single group assignment (iii) Masking: double (participant, outcomes assessor) (iv) Primary purpose: treatment Outcome measures: (v) Mobility-6 min self-paced walk (vi) Mobility-sit to stand (vii) Mobility-static balance (viii) Mobility-6 min fast-paced walk (ix) Intermittent and constant knee pain (x) Energetics-submaximal oxygen consumption (xi) Energetics-maximal oxygen consumption (xii) Energetics-hydrogen peroxide emission (xiii) Bone mineral density (xiv) Knee range of motion (xv) Leg extensor strength	NCT04638387

## Data Availability

The data used to support the findings of this study are available from the corresponding author upon request.
